# Human papilloma virus (HPV) prevalence upon HPV vaccination in Swedish youth: a review based on our findings 2008–2018, and perspectives on cancer prevention

**DOI:** 10.1007/s00404-020-05879-7

**Published:** 2020-11-27

**Authors:** Juan Du, Andreas Ährlund-Richter, Anders Näsman, Tina Dalianis

**Affiliations:** 1 Department of Microbiology, Tumor and Cell Biology, Centre for Translational Microbiome Research (CTMR), Karolinska Institutet, Stockholm, Sweden; 2 Department of Oncology-Pathology, Karolinska Institutet, Stockholm, Sweden

**Keywords:** HPV prevalence, HPV vaccines, Cervical cancer, Tonsillar cancer, Base of tongue cancer, Screening

## Abstract

**Purpose:**

Three human papillomavirus (HPV) vaccines are available against up to nine HPV types. In Sweden, from 2012, Gardasil was offered to 10−12 year old girls through the school-based vaccination program, and as catchup vaccination for women up to 26 years. To obtain a baseline, and follow HPV vaccination effects, during 2008−2018, cervical and oral HPV prevalence were followed at a youth clinic in Stockholm, and in 2013 for comparison oral HPV prevalence was examined in high-school youth in a middle-sized county in Sweden.

**Methods:**

In this review, we discuss all our data with cervical and oral mouthwash samples that were collected and tested for 24−27 HPV types by a bead-based multiplex assay from 2008.

**Results:**

Compared with 2008−2011, with ~ 35% HPV16 and > 60% high risk (HR) HPV cervical prevalence at the youth clinic, a decrease of vaccine HPV types was observed between 2013 and 2018, with e.g., HPV16 falling to 5% in catchup vaccinated women and 15−18% in nonvaccinated women. Most common cervical HR-HPV types were HPV39, 51, 52, 56, and 59 together accounting for ~ 10% of cervical cancer, and where only HPV52 is included in Gardasil-9. At baseline 2009−2011, oral HPV prevalence was ~ 10% in unvaccinated youth at the youth clinic, but after 2013 it dropped to < 2% at the youth clinic and high schools.

**Conclusion:**

To conclude, Gardasil HPV types have decreased, but it is still important to follow remaining HR-HPV types and cancer development, since there is an ongoing increase in the incidence of HPV-associated tonsillar and base of tongue cancer, and cervical cancer in Sweden.

## Introduction and historic perspective

In 1983, when HPV16 was finally disclosed in cervical cancer, this finding was complex since several HPV types were being discovered and found to cause cervical cancer [1–5].

Today, > 200 HPV types have been discovered, of which most are cutaneous and ~ 40 mucosal and defined as low risk (LR), putatively high risk (HR), or HR−HPV types [5]. All have an ~ 8 kb double-stranded circular DNA genome encoding the E1−E2, E4−E7 regulatory proteins. E6 and E7 of HR-HPV types, binding p53 and retinoblastomprotein (Rb) and dysregulating the cell proliferation, are classified as oncogenes [5]. L1 and L2 are structural proteins, form the viral capsid, and L1 capable of forming virus like particles (VLPs) is the basis for HPV vaccines [5].

In 1995, HPV16 was recognized as a carcinogen in cervical cancer by the International Agency for Research against cancer (IARC) [6]. Oropharyngeal squamous cell carcinoma (OPSCC), and its subsites tonsillar and base of tongue cancer (TSCC/BOTSCC) were reported to be associated with HPV16 infection in 2000 and 2007, respectively. This was also recognized by the IARC [8–10] By then, TSCC and BOTSCC were increasing drastically in many Western countries, as has increased in Sweden [11, 12].

In parallel, in 2006, the first HPV vaccine Gardasil, which against HPV16, 18, 6 and 11, was approved by the Food and Drug Administration. This was the case for Cervarix (against HPV16 and 18) and Gardasil-9 (against HPV16, 18, 31, 33, 45, 52, 58, 6 and 11) in 2009 and 2014, respectively [7].

This review presents details of the period during the introduction of the HPV vaccine with focus on young individuals at a youth clinic in Stockholm, Sweden, but also in youth at high schools in a median-sized county in Sweden (Table 1). In addition, parallel studies on possible interplay of vaginal microbiota and HPV infection, and perspectives on precancerous lesions and screening are briefly commented on.

**Table 1:** Overview of the studies included in this review

Collection period	Place	Sample size^a^	Vaccine%^b^	Sample type	HPV%	Top five HR-HPV types^c^	Reference number
2008−2010	Youth clinic	544	0	Cervical samples	70%	16,51,18,52,73	14
2009−2011	Youth clinic	483	0	Oral samples	9.3%	16,59,51, (56,82)	15
2009−2011	Youth clinic	174	0	Cervical samples	74%	16,52,51,18, (56, 59)	15
2013	High school	335	64% in 160 females	Oral samples	1.8%	16, (56, 68)	17
2013−2014	Youth clinic	211	73%^a^	Cervical samples	61% in vaccinated & 70% in nonvaccinated women	51, (56, 59, 73), 39 in vaccinated women & 16, (51, 56, 39), (59, 52, 45, 31) in nonvaccinated women	18
2013−2014	Youth clinic	287	73% in 200 females	Oral samples	1.4%	16, (51,52,59)	18
2013−2015	Youth clinic	338	71%	Cervical samples	65% in vaccinated & 75% in nonvaccinated women	56, (59, 51), (52, 39, 73) in vaccinated women & 59, 16, 52, (51, 73, 82) in nonvaccinated women	19
2013−2015	Youth clinic	457	71% in 335 females	Oral samples	1.5%	(16, 51, 52, 59, 39, 42, 35)	19
2017−2018	Youth clinic	172	82%	Cervical samples	67% in vaccinated & 93% in nonvaccinated women	56, 59, 51, 52, 39, in vaccinated women & (56, 51), (52, 39), (73, 33, 16, 45) in nonvaccinated women	20

^a^Samples with enough material; Oral samples were collected from both genders

^b^Most of the women got catchup vaccination

^c^More HPV types are listed in () if the prevelence is same

## HPV vaccination in Sweden and background to our studies

HPV vaccination was gradually introduced in Sweden between 2006−2011. In 2012, Gardasil was supplied for 10−12 year old girls through the school-based vaccination program, and catchup vaccination of girls aged 13−26 years was also offered for free, depending on Swedish county [13]. From September 2019, Gardasil-9 has been used in the school-based vaccination program, and this autumn 2020, boys 10−12 years of age start to be vaccinated in Sweden.

Prior to the introduction of HPV vaccination, we had described HPV causative of TSCC/BOTSCC and reported increasing incidences of HPV-positive TSCC and wanted to follow vaccination effects on oral HPV prevalence [11]. Therefore, we collaborated with the Swedish Agency for Infectious Disease Control and examined cervical and oral HPV prevalence in youth aged 15−23 years at a large youth clinic in Stockholm, Sweden. Investigations conducted during 2008−2010 and 2009−2011 obtained a baseline for cervical and oral HPV prevalence (later found ~ 70% and ~ 10% respectively) before HPV vaccination and compared HPV types at the two locations [14, 15]. The latter was of interest, due to that e.g., HPV16 accounted for ~ 90% of all HPV positive TSCC/BOTSCC as compared to ~ 55% of all cervical cancer [11, 16].

In 2013, with colleagues in Uppsala, we performed a study on life style and oral HPV prevalence, in high school classes in a middle-sized county in Sweden, and reported that oral HPV prevalence was low, but by then almost 70% of the young girls had been catchup HPV vaccinated [17]. This prompted us to do follow-up studies 2013−2018 at the youth clinic in Stockholm where changes in cervical and oral HPV prevalence were observed, with decreases in HPV vaccine types, but persistence of HPV types not covered by the vaccines [18–20].

## The youth clinic, high schools, the study designs, sample collection, and HPV typing

### The youth clinic

The first two studies partly overlapped and were conducted 2008−2010 and 2009−2011 in one of the largest youth clinics in Stockholm, visited annually by 4000 women and 800 men aged 15−23 years, for advice on birth control and treatment of sexually transmitted diseases [14, 15]. The three follow-up studies were performed 2013−2018 [18–20]. The youth were from the local area and the universities of Stockholm. Participation was voluntary, anonymous, and performed after ethics approval. The data on birth year and HPV vaccination status were acquired for all, and for microbiota studies, information on antibiotic treatment was added. In the two first studies, the basis of HPV prevalence data was analysed on only unvaccinated individuals, and the proportion of HPV-catch up vaccinated individuals was ~ 10−20% [14, 15]. In the follow up studies, HPV prevalence data was acquired from both HPV nonvaccinated and catchup vaccinated individuals, and by then ~ 70% were catchup vaccinated, and with time subsequently some were vaccinated through the school-based vaccination program [18–20]. The participation rate was ~ 15% due to the work load of the midwives, who could inform the youth and collect samples maximum 1−2 times per week [14]. Roughly 1200 youth have participated in our studies over the years [14, 15, 18–20].

### High schools

Youth from their first year (877 students) at 13 senior high schools from one municipality with a population of 140,000 in mid-south Sweden, initially participated in a study on health, life style, sexual experiences, attitudes towards sexuality, and pornography consumption. During their third year, a follow up study was conducted, with 627 participants contributed a voluntary collection of mouthwash samples. Among those, 335 students (160 females and 175 males), aged 17–21 years (median age 18 years) donated oral samples for our study, with collection initiated in January 2013 and completed in March 2013 [17].

### Sample collections

Oral samples were obtained by a gargle for 30 s with 15 ml 50% Listerine (Johnson and Johnson Consumer Nordic) in plastic containers (Sarstedt, 25-ml screw cap yellow, Sweden), stored at + 4 °C for 0−3 days, transported back to the lab and treated as described before [15, 17]. Cervical samples were collected either by a midwife or by self-collectiom using nylon flocked swabs (Copan, Brescia, Italy), and preserved in sterile tubes containing 5 ml SurePath preservation solution (BD, Sweden) or 0.8 ml DNA/RNA shield (Zymo Research, CA, USA), and stored at + 4 °C before treatment [14].

### HPV testing

Samples were initially tested for 24 and later 27 mucosal HPV types using L1 primers. Extra E6 primers for HPV16 and HPV33, and β-globin gene primers as controls were also analysed by a Luminex-based multiplex assay, as described before [14–17]. HR-HPV or LR-HPV types were classified according to Muñoz et al., 2006 [21]. Median fluorescence intensity (MFI) > 30 for β-globin was defined as adequate DNA quality and data interpretation. Samples with MFIs > 8 + 1.5 × background were considered HPV positive [14].

## Initial HPV prevalence studies 2008−2011, at a large youth clinic in Stockholm, Sweden

### The two initial studies at the youth clinic

The 1st study (2008−2010) initially included 544 nonvaccinated women donating cervical samples, while the 2nd study (2009−2011) included 408 women and 82 men donating oral samples, where 180 of the women had also donated cervical samples [14, 15]. During this period ~ 10% of the women had been HPV catchup vaccinated and samples from these women were not included in the analysis [14] (Table 1).

### Cervical prevalence

Cervical HPV prevalence in young women was similar in the 1st and 2nd studies, i.e., 70 and 74%, respectively, of the samples tested positive for one or more HPV types, of which most were HR types (> 60%), with HPV16 dominating, with 34.7% and 37.9%, respectively [14, 15] (Table 1). Other common HR types were HPV52, 51 and 18 at roughly 10−15%. The most common LR type was HPV42 (~ 17%), while LR HPV6 and 11 included in the vaccines were less common (around 8 and 2%, respectively). The highest cervical HPV prevalence was in women aged 19−21 years.

### Oral prevalence

Oral HPV prevalence in young men and women was similar with 9.3% for the whole group, 9.8% in men and 9.2% in women (Table 1). HR-HPV types accounted for 7.2% of all oral samples, and HPV16 dominated with 2.9%, while HPV18 was only detected in one sample (0.2%) [15]. Notably, oral HPV prevalence was high in women with cervical HPV infection (17.1%) as compared to those with no cervical infection (4.4%) (*p* = 0.043) [15].

### Oral and cervical prevalence showed concordance in the cervix and oral cavity

In the 2nd study, we observed that MFI signals in the cervix were higher, and many more HPV types were generally found in the cervix as compared to the oral cavity, likely due to that in the latter 0.5−1.5 L/day of saliva is produced. Notably, all HPV types detected in the oral cavity were also observed in the cervix, but not vice versa [15].

## Oral HPV prevalence among high school students at a medium-sized county in Sweden

The studies conducted in youth at the youth clinic, were suggested not to be representative of youth in general, since the former may have been more sexually active, and many visited for checking for sexually transmitted diseases.When compared with international reports, both cervical and oral HPV prevalence at the youth clinic in Stockholm was relatively high [14, 15, 22, 23]. It was therefore valuable to examine HPV prevalence in a high school setting. Colleagues in Uppsala, were conducting a life style study among high school students, so we joined forces after obtaining ethical permission. Mouth wash samples were collected from 160 females and 175 males (335 in total) high-school students, aged 17−21 years (median 18 years of age). According to the questionnaire, 64% of the females had been catchup vaccinated against HPV at a median age of 16.4 years, while the median age for sexual debut was 15.2 years. Notably, the overall oral HPV prevalence in this setting was 1.8%, with oral HR-HPV prevalence at 3.1% (5/160) in females (all 18 years of age) and 0.6% (1/175) in males (19 years of age) (Table 1). Among the females, 4/5 HPV infections were with HPV16, and one had an HPV58 infection, and the only male had an oral HPV56 infection. Of the girls 4/5 were HPV vaccinated, between 2009 and 2012, but all had been vaccinated after their sexual debut. The oral HPV prevalence of 1.8% was much lower than that at the youth clinic 2009−2011, where only few individuals had been vaccinated and we only had baseline data [17]. Therefore, we returned to the youth clinic, to examine possible changes in HPV prevalence there.

## Continued studies 2013−2018, on HPV prevalence at the Stockholm youth clinic

Three studies after each other were conducted, 2013−2015, 2013−2016, and 2017−2018. All three documented similar changes, and the HPV catchup vaccination rate was > 70% [18–20].

### Cervical prevalence

**Fig. 1:**
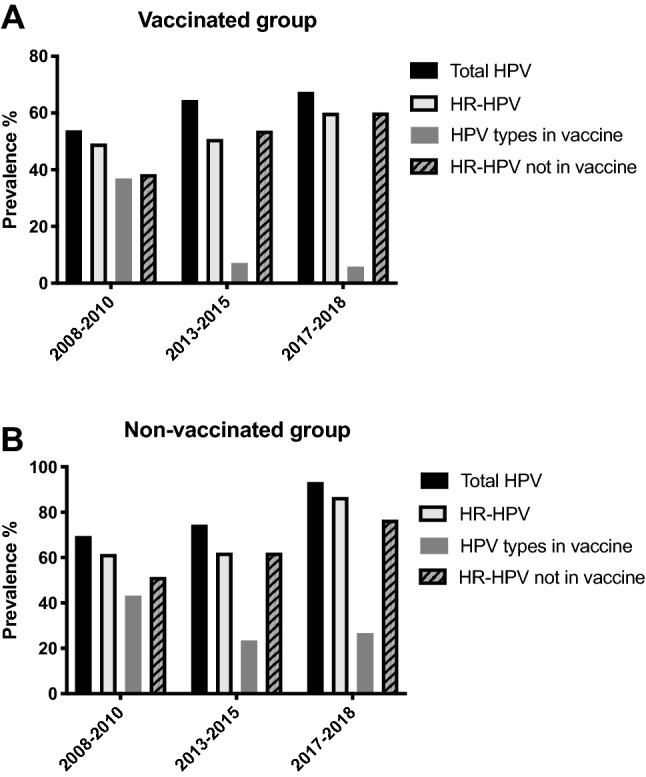
Cervical HPV prevalence in youth during 2008−2010, 2013−2015, and 2017−2018 at a youth clinic in Stockholm. The data are presented as total HPV prevalence, HR-HPV prevalence, HPV types in the tetravalent vaccine, and HR-HPV types not covered in vaccine among HPV vaccinated (most of the women got catchup vaccination) (**a**) and non-HPV vaccinated youth (**b**)

Cervical prevalence was in these three follow-up studies examined in roughly an additional 500 women (Table 1) [18–20]. The prevalence of HPV16 had dropped to virtually 5% in catchup HPV vaccinated women, and to roughly 18% in unvaccinated women, as compared to that reported previously (34.1−37.8%) [14, 15, 18–20]. Likewise, the cervical prevalence of all HPV vaccine types, when grouped together, had decreased considerably in catchup vaccinated young women, as well as to some extent in nonvaccinated women, while nonvaccine HPV types had remained and were increasing relatively (Fig. 1) [20]. It was also of note that in the latter study 2017−2018, that the youngest girls (16−17 years of age) had been vaccinated through the school-based program and they exhibited extremely low prevalence of the HPV vaccine types [20]. Nonetheless, the frequency of HR-HPV infections of which most were not included even in Gardasil-9, remained high and these account for around 10% of cervical cancer in the same region during the time period 2003–2008 [16, 20].

### Oral prevalence

Oral HPV prevalence also dropped considerably to 1.5% (7/457) when assessed at the youth clinic in the study of 2013−2015 (Table 1) [19]. Seven samples in total were HPV positive, 3 from men and 4 from women, with only one being positive for HPV16. Thus, there was a prominent decrease in the oral HPV prevalence in Stockholm as well [15, 17].

### Cervical and oral HPV prevalence the past 10 years

To conclude, at the youth clinic both cervical and oral HPV prevalence were fairly high from an international perspective, and in comparison to the relatively few reports that existed during similar periods [16, 17, 24]. However, upon HPV catchup vaccination, HPV prevalence of the HPV vaccine types decreased considerably, although leaving HR-HPV types still there [22, 25]. This means that it is important to continue with screening of women for cervical cancer.

## Parallel similar studies of the impact of HPV vaccination on cervical HPV prevalence

In a recent systemic review and meta-analysis, the data from 60 million individuals were covered in 65 articles from 14 high-income countries, and of these 65 articles, 23 followed HPV infection, 29 followed anogenital warts, and 13 followed CIN2 + [26]. The meta-analysis showed compelling evidence of a significant impact of three-dose girls-only HPV vaccination programs, with the tetravalent Gardasil and bivalent Cervarix vaccines, on infections with HPV16 and 18, and HPV31, 33, and 45 as a group. This was also the case for anogenital warts and CIN2 + . The researchers also found evidence of herd immunity. Basically, our data with regard to decreases of the HPV vaccine types in cervical samples of catchup vaccinated women, as well as potential herd immunity in nonvaccinated women are very much in line with the compiled data of the meta-analysis [27]. The data on the effects of HPV vaccines on oral HPV infections are limited. However, a recent European study shows data very similar to ours, with a decrease in oral HPV prevalence after HPV vaccination, as also shown earlier in Costa Rica [27, 28].

## Future perspective

### Screening of cervical cancer

Screening of cervical cancer is according to the National Board of Health and Welfare today recommended, by cytology for women 23−29 years every third year, by HPV testing for women 30−49 years every 3rd year, and by complementary cytology at the age of 41 years [12]. For women 50−64 years of age, HPV testing is done every 7 years. In our opinion, it may be of importance to have both L1 and E6 primers for all HR-HPV types in HPV testing, since it has been documented that L1 can often be lost upon cancer development [5]. Furthermore, importantly, not all HR-HPV types have yet disappeared [20].

### Screening for HPV-positive TSCC and BOTSCC

As mentioned above, TSCC and BOTSCC are increasing in incidence, and possible screening has been discussed. However, the presence of oral HPV infection is relatively rare, likely due to the secretion of saliva in the oral cavity. Furthermore, others and we have shown that the presence of HPV in the oral cavity is not necessarily associated with the presence of HPV in the tonsils, and therefore not necessarily correlated to cancer development [24]. Moreovers, some researchers claimed that there are no pre-stages of OPSCC [25].

Nonetheless, we have shown that patients with HPV-positive TSCC and BOTSCC often have high MFI levels of the tumour HPV type in their oral cavity upon donating a gargle sample, and these levels are roughly 200 times higher than the HPV MFIs in oral rinses of healthy youth [15, 29]. It was also occasionally possible to disclose cytological changes in oral rinses of TSCC and BOTSCC patients [29]. A very high MFI in a mouth wash could be indicative of a risk for TSCC/BOTSCC; however, it has not been proved as a reliable diagnostic indicator for TSCC/BOTSCC. Likewise, it has been reported that antibodies against  E6 can develop > 10 years before development of an oropharyngeal cancer, but this has not been shown to work on an individual level neither [30].

Notably, recently attempts have also been made to look at pre-stages of TSCC and BOTSCC, and we have also compared dysplastic lesions in TSCC to invasive cancer [31]. Forty genes were shown to be differentially regulated between HPV-positive dysplastic tissue and invasive cancer tissue, and 33 genes from HPV-negativedysplastic tissue and invasive cancer tissue. Five of the nine most affected pathways showed similar increased activity in both HPV-positive and negative invasive cancer as compared to dysplastic lesions. Taken together, the data suggest that both HPV-positive and HPV-negative TSCC/BOTSCC precancerous lesions do exist. Nevertheless, further refinement could be possible, such as using some of the most prominent markers, e.g., SPARC, psoriasin, type I collagen, and galectin-1 in future blood tests for cancer screening [31].

### HPV and microbiota

Vaginal microbiota has recently been linked to an increased risk of sexually transmitted infections, including HPV infection [32]. Our data are also supported by others, showing that vaginal microbiota dominated by non-*Lactobacilli* species or *Lactobacillus iners* were associated with three to five times higher odds of any prevalent HPV and two to three times higher for high-risk HPV and dysplasia/cervical cancer compared with *Lactobacillus crispatus* [33]. Meta-analysis on vaginal microbiota and the risk of HPV and cervical cancer also supported a causal link between vaginal dysbiosis and cervical cancer along with HR-HPV acquisition, persistence, and cervical dysplasia development [34]. A potential relationship of certain microbiota with a faster clearance of HPV infection has been suggested, but this field needs to be investigated further with larger and logituginal corhorts. If this is the case maybe one could abrogate a persistent HPV infection by modifying the vaginal microbiota. This of course would be very valuable information and be very helpful in getting rid of persistent HPV infection.

## Concluding remarks

To summarize, presentation of HPV vaccination in the school-based program and as catchup vaccination has successfully decreased the cervical and oral HPV prevalence of the Gardasil HPV16, 18, 6, and 11 types, both in vaccinated and nonvaccinated youth. Continued HPV vaccination on both genders would be extremely valuable and allow for a further decrease in HPV vaccine types and their associated cancers [35].

Nonetheless, other HR-HPV types still persist, of which most are not included in Gardasil-9 and these accounted for 10% of all cervical cancer in the Stockholm region 2003−2008. Therefore, it is important to follow-up patients with cervical screening, and investigate which HR-HPVs are present in HPV-associated tumors in the future.

In addition, more knowledge on microbiota may allow for better means of combating persistent HPV infections. Future cancer screening could also use the knowledge acquired on different protein expression in dysplastic and invasive lesions of TSCC and BOTSCC.
